# Pregnancy bias toward boys or girls: The Japan Environment and Children’s Study

**DOI:** 10.1371/journal.pone.0287752

**Published:** 2023-06-23

**Authors:** Shuhei So, Fumiko Tawara, Yu Taniguchi, Naohiro Kanayama, the Japan Environment and Children’s Study (JECS) Group

**Affiliations:** 1 Department of Reproductive and Perinatal Medicine, Hamamatsu University School of Medicine, Shizuoka, Japan; 2 Tawara IVF Clinic, Shizuoka, Japan; 3 Japan Environment and Children’s Study Programme Office, National Institute for Environmental Studies, Tsukuba, Japan; 4 Department of Obstetrics and Gynecology, Hamamatsu University School of Medicine, Shizuoka, Japan; University of Abuja Teaching Hospital, NIGERIA

## Abstract

The sex of the conceived child is a significant concern for parents. To verify whether there women have pregnancy bias toward boys or girls, we investigated whether the history of continuous same-sex pregnancy was associated with the subsequent child’s sex. We prospectively analyzed data from the Japan Environment and Children’s Study, a birth cohort study. We included all cases of singleton live births (n = 98 412). Women with pregnancy due to infertility treatment were excluded (n = 6255); Similarly, women with a history of miscarriage, artificial abortion, stillbirth, and multiple pregnancies, and those with missing data on the sex of the previous child were excluded. Altogether, 62 718 women were included. For the first live birth, a male-biased sex ratio of 1.055 was observed. Further, no significant difference was found in the sex ratio of the conceived child between women with one boy and those with one girl previously. However, when there were more than two children previously, the subsequently conceived child’s male/female sex ratio was significantly higher among boy-only mothers than among girl-only mothers. The results indicated that several pregnant women are biased toward conceiving either boys or girls.

## Introduction

According to Fisher’s principle, the sex ratios of several organisms are balanced at 1:1 [1]. Moreover, the human male/female sex ratio is approximately 1:1 but reportedly has a slight male bias [1, 2]. Results from several studies have shown that the male bias in the secondary sex ratio (SSR) is due to the male bias in the primary sex ratio (PSR) at conception [1, 3, 4]. In contrast, Orzack et al. argued that the SSR becomes male-biased due to more pregnancy losses of girls than boys based on a meta-analysis of the sex ratios in chorionic villus sampling, amniocentesis, induced abortion, stillbirth, and live births [5]. Additionally, female-selective abortions lead to SSR imbalances in several countries [6]; thus, SSR contributes to both sex-biased pregnancy and loss.

To determine if there are women with pregnancy bias towards boys or girls, we investigated whether the history of continuous same-sex pregnancy was associated with the subsequent child’s sex.

## Materials and methods

### Study design

We analyzed data from the Japan Environmental Children’s Study (JECS), a birth cohort study undertaken to elucidate the influence of chemical exposure during the fetal period and early childhood on children’s health, with follow-up until the age of 13 years. This study’s protocol and baseline data are available elsewhere [7, 8]. The JECS is funded directly by Japan’s Ministry of the Environment and involves collaboration between the National Institute for Environmental Studies, the National Centre for Child Health and Development, and 15 Regional Centers. Pregnant women were recruited between January 2011 and March 2014. Eligibility criteria for participants (expectant mothers) were as follows: 1) residing in the study areas at the time of recruitment and enrolled with co-operating health care providers and local government offices issuing Maternal and Child Health Handbooks; 2) expected delivery date after 1 August 2011; and 3) capable of comprehending the Japanese language and completing the self-administered questionnaire.

### Ethical approval

The JECS protocol was reviewed and approved by the Ministry of the Environment’s Institutional Review Board on Epidemiological Studies and the Ethics Committees of all participating institutions (No.100910001). The JECS was conducted in accordance with the principles of the Declaration of Helsinki and other internationally valid regulations and guidelines. Written informed consent was obtained from all the participants.

Personal information is managed strictly by limiting the number of people who can handle it. Biospecimens and questionnaires will be kept after removing any personally identifiable information. Since the research is conducted using only the reference number, the researcher will not be able to tell who the work belongs to.

### Sample selection

**Fig 1:**
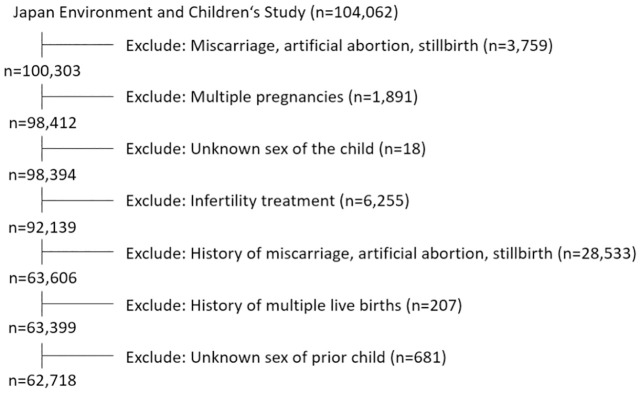
Flow chart for the selection of participants from the Japan Environmental Children’s Study.

This study was based on the ‘jecs-ta-20190930’ data set, which was released in October 2019. The data set included 104 062 records, and we included all cases of singleton live births (enrolled participants, n = 98 412). Further, cases with pregnancy due to infertility treatment were excluded because infertility treatments affect the sex ratio of children due to procedural intervention (enrolled participants, n = 92 139). In addition, women with a history of miscarriage, artificial abortion, and stillbirth, including those with a history of multiple pregnancies and missing data on the sex of the previous child, were.excludes. Finally, 62 718 women were included in the study (Fig 1).

### Variables

Data transcribed from medical records at the time of study enrollment included information on maternal age, history of multiple pregnancies, miscarriage, artificial abortion and stillbirth, sex of siblings, use of in vitro fertilization, and embryo transfer (IVF-ET) for the present pregnancy. Data transcribed from medical records immediately after delivery included maternal and gestational age, single/multiple pregnancy status, infant sex, and pregnancy outcomes such as live/stillbirth and miscarriage/induced abortion.

### Statistical analyses

The differences between the two groups were assessed using Pearson’s chi-square test. Statistical analyses were performed using JMP14 software (SAS Institute Inc., Cary, NC, USA). Multiple testing was adjusted for using the Bonferroni method.

## Results

In this study, 62 718 women without a history of miscarriage, artificial abortion, and stillbirth were enrolled. For the first live birth, a male-biased sex ratio of 1.055 was observed (Table 1). Subsequently, no significant difference was found in the sex ratio of the conceived child between women with one boy previously and those with one girl previously (1.068 vs. 1.039, *p* = 0.290, Table 1). However, when there were more than two children previously, the subsequently conceived child’s male/female sex ratio was significantly higher among boy-only mothers than among girl-only mothers (1.112 vs. 0.972, *p* = 0.014, Table 1). As a subgroup analysis, the subsequently conceived child’s male/female sex ratios of mothers with two, three, and four boys children are higher than those of girl-only mothers in each case, although no significant difference is observed (1.100 vs 0.987 for two prior children, p = 0.058; 1.169 vs 0.824 for three prior children, p = 0.075; 1.750 vs 0.750 for four prior children, p = 0.167, Table 2).

**Table 1:** Relationship between the sex of a child in the current pregnancy and sex of prior children of the participants.

Number of children from previous pregnancies	Sex of children from previous pregnancies	n	Sex of the subsequent child		M/F	*p*	Relative risk [97.5% CI] (Male/Female)
			Male	Female			
None	-	27 874	14 313	13 561	1.055	-	-
One	A boy	12 361	6385	5976	1.068	0.290	1.01[0.99–1.04]
	A girl	11 419	5820	5599	1.039		
Two or more	Only boys	2 990	1574	1416	1.112	0.014	1.07[1.01–1.13]
	Only girls	2 433	1199	1234	0.972		

The differences between the two groups were assessed using the Pearson chi-square test. P-value and Relative risk [97.5% CI] are adjusted by Bonferroni correction; P<0.025 (p = 0.05/2) indicates statistically significant results. CI, confidence interval; M, male; F, female

**Table 2:** Subgroup analysis of women with more than two children.

Number of children from previous pregnancies	Sex of children from previous pregnancies	n	Sex of the subsequent child		M/F	*p*	Relative risk [98.3% CI] (Male/Female)
			Male	Female			
Two	MM	2686	1407	1279	1.100	0.058	1.05[0.99–1.13]]
	FF	2211	1098	1113	0.987		
Three	MMM	269	145	124	1.169	0.075	1.19[0.95–1.50]
	FFF	197	89	108	0.824		
Four	MMMM	33	21	12	1.750	0.167	1.48[0.75–2.93]
	FFFF	21	9	12	0.750		

The differences between the two groups were assessed using the Pearson chi-square test. P-value and Relative risk [98.3% CI] are adjusted by Bonferroni correction; P<0.017 (p = 0.05/3) indicates statistically significant results. CI, confidence interval; M, male; F, female

Women with five children or more were excluded due to the small number of cases: women with five children, MMMMM (n = 2, one boy and one girl in the subsequent child), FFFFF (n = 3, two boys and one girl in the subsequent child). Women with six children, FFFFFF (n = 1, one boy in the subsequent child).

## Discussion

Our study used a large Japanese database involving 62 718 early pregnant women without a history of miscarriage, artificial abortion, and stillbirth and found that women with more than two prior boys or girls were significantly more likely to have another boy or girl, respectively. Biggar et al. conducted an epidemiological study to explore the relationship between sex ratio and birth order using the Danish National Database, which records the sex and birth order of 1.4 million infants [6]. Although 51.2% of the first-birth children were boys, the probability of having another boy increased to 51.5%, 51.6%, 52.4%, and 54.2% for women who had previously conceived one, two, three, or four boys, respectively, suggesting the existence of families with child’s sex-biased toward boys. Although it was unclear whether such sex bias resulted from male-biased pregnancy or female-biased pregnancy loss, our findings suggest that the SSR bias toward either a boy or girl may be due to inherent sex bias upon subsequent pregnancy rather than sex bias due to pregnancy loss.

The sex ratio of a fertilized egg depends on the sex chromosomes carried by the spermatozoa. Most reports have shown no difference in the frequency of Y- and X-bearing spermatozoa [9–12]. Interestingly, Umehara et al. found that Toll-like receptors (TLR7/8) were expressed in the X-bearing spermatozoa in mice, and their active state showed decreased motility of the X-bearing spermatozoa [13]. This result suggested that males consuming an anti-viral compound, which is a ligand for TLR7/8, have decreased motility of X-bearing spermatozoa and may have increased XY embryo production. Males with a history of hepatitis B virus infection have been reported to have a high male/female sex ratio [14]. Differences were noted in motility, viability, electrophobicity, pH susceptibility, surface properties, and stress response between the X- and Y-bearing spermatozoa [4, 15]. These factors related to sperm activity will not only be affected by the testis and seminiferous tubules as male factors but also by the female vaginal environment. Thus, as an example, the sex bias of the activated sperm may be involved in the SSR.

This study has some limitations. First, since it was conducted using a Japanese database, almost all participants were Japanese, limiting the generalizability to other populations. Second, although some environmental factors might influence the sex ratio, we did not investigate the relationship between sex-biased pregnancies and these confounding factors, presuming that the environmental factors during the survey and the environment during the previous pregnancies differed. Third, it is unknown whether all children involved were born to the same father in all mothers’ pregnancies. Finally, we did not examine the relationship between sex bias upon subsequent pregnancy and paternal factors, including age and medical history.

In conclusion, mothers with a pregnancy history of consecutive boy or girl children were more likely to be biased toward boys or girls, respectively, for the sex of the subsequently conceived child. This epidemiological study will provide crucial insights into women who become pregnant with a bias for boys or girls. Further studies are required to clarify whether sex-biased pregnancy is influenced by PSR or conception and whether it is affected by paternal or maternal factors.
